# Increased Levels of ER Stress and Apoptosis in a Sheep Model for Pulmonary Fibrosis Are Alleviated by *In Vivo* Blockade of the KCa3.1 Ion Channel

**DOI:** 10.1155/2021/6683195

**Published:** 2021-03-19

**Authors:** Udari E. Perera, Louise Organ, Sasika N. V. Dewage, Habtamu B. Derseh, Andrew Stent, Kenneth J. Snibson

**Affiliations:** ^1^School of Veterinary Science, The University of Melbourne, Parkville, VIC, Australia; ^2^Nottingham Respiratory Research Unit, University of Nottingham, Nottingham, UK

## Abstract

Idiopathic pulmonary fibrosis (IPF) is a fatal interstitial lung disease, characterized by progressive damage to the lung tissues. Apoptosis and endoplasmic reticulum stress (ER stress) in type II alveolar epithelial cells (AECs) and lung macrophages have been linked with the development of IPF. Therefore, apoptosis- and ER stress-targeted therapies have drawn attention as potential avenues for treatment of IPF. The calcium-activated potassium ion channel KCa3.1 has been proposed as a potential therapeutic target for fibrotic diseases including IPF. While KCa3.1 is expressed in AECs and macrophages, its influence on ER stress and apoptosis during the disease process is unclear. We utilized a novel sheep model of pulmonary fibrosis to demonstrate that apoptosis and ER stress occur in type II AECs and macrophages in sheep with bleomycin-induced lung fibrosis. Apoptosis in type II AEC and macrophages was identified using the TUNEL method of tagging fragmented nuclear DNA, while ER stress was characterized by increased expression of GRP-78 ER chaperone proteins. We demonstrated that apoptosis and ER stress in type II AECs and macrophages increased significantly 2 weeks after the final bleomycin infusion and remained high for up to 7 weeks post-bleomycin injury. Senicapoc treatment significantly reduced the rates of ER stress in type II AECs and macrophages that were resident in bleomycin-infused lung segments. There were also significant reductions in the rates of apoptosis of type II AECs and macrophages in the lung segments of senicapoc-treated sheep. In vivo blockade of the KCa3.1 ion channel alleviates the ER stress and apoptosis in type II AECs and macrophages, and this effect potentially contributes to the anti-fibrotic effects of senicapoc.

## 1. Introduction

Idiopathic pulmonary fibrosis (IPF) is a fatal interstitial lung disease characterized by progressive damage to the lung tissues, which in turn leads to the impairment of the gas exchange function of the lungs [1–3]. While pirfenidone and nintedanib are two drugs currently approved by the FDA for slowing the progression of IPF [4–7], these drugs do not cure the ailment [4–7]. Investigation of potential therapeutic targets to inhibit the ongoing fibrosis remains a major challenge due to a lack of understanding of the pathogenesis of fibrosis in the lungs.

The progression of IPF involves many disease elements including activation of transforming growth factor *β* (TGF-*β*) and other chemokines, type II alveolar epithelial cell (AECs) apoptosis, ineffective removal of apoptotic cells, and aberrant wound healing and endoplasmic reticulum (ER) stress [8, 9]. The ER is a special organelle normally responsible for the folding synthesized proteins and subsequent vesicular transport of proteins to the Golgi apparatus. Factors such as Ca^2+^ depletion, redox homeostatic alteration, nutrient deprivation, and environmental insults can affect these folding processes, eventually leading to accumulation of unfolded proteins which disrupt the ER function, resulting in ER stress [10].

ER stress is known to initiate apoptosis pathways [11] through activation of caspase-12. Caspase-12 enables specific cleavage of caspase-9, which together with downstream effects eventually leads to apoptosis [11].

ER stress in type II AECs is one of the prominent features of IPF and is frequently found in patients with familial and sporadic IPF [12]. ER stress has also been observed in macrophages in the lungs of patients with asbestosis [13]. ER stress induces inflammatory signaling, epithelial-mesenchymal transition, myofibroblast activation, macrophage polarization, and T cell differentiation that may contribute to the disease progression of IPF [14–17].

The calcium-activated potassium ion channel KCa3.1 has been proposed as a potential therapeutic target for fibrotic diseases including IPF [18–21]. The KCa3.1 ion channel is expressed in a number of cell types in the lungs and other tissues [18, 20, 22]. Importantly, stimulation of the KCa3.1 ion channel activates cells such as fibroblasts, macrophages, and epithelial cells that are involved in the disease processes of IPF [18]. We have recently reported that blockade of the KCa3.1 ion channel using senicapoc (ICA-17043) attenuates interstitial lung fibrosis, improves lung compliance, and attenuates microvascular remodeling in a sheep model for pulmonary fibrosis [23, 24]. In the current study, we utilized the sheep model to analyze the regulatory function(s) of the KCa3.1 ion channel in type II AECs and macrophages in fibrotic disease.

Our aims in the present study are to firstly ascertain whether ER stress and increased rates of apoptosis in type II AECs and macrophages are observed in sheep lungs with experimentally induced fibrosis. Secondly, we investigate whether blockade of KCa3.1 ion channel with a specific inhibitor, senicapoc, will retard ER stress and the rate of apoptosis in type II AECs and macrophages in this large animal model of pulmonary fibrosis.

## 2. Materials and Methods

### 2.1. In Vivo Experimental Design to Evaluate ER Stress and Apoptosis in Type II AEC and Macrophages in Bleomycin-Induced Lung Fibrosis Using Sheep Model

All experimental procedures relating to sheep experiments and sample collection were approved by Animal Experimentation Ethics Committee, University of Melbourne (Parkville, VIC, Australia) and adhere to the Australian Code of Conduct for the Care and Use of Animals for Scientific Purposes. Sheep lung tissue samples were obtained from another study [25] and the experimental design was briefly as follows.

**Figure 1:**
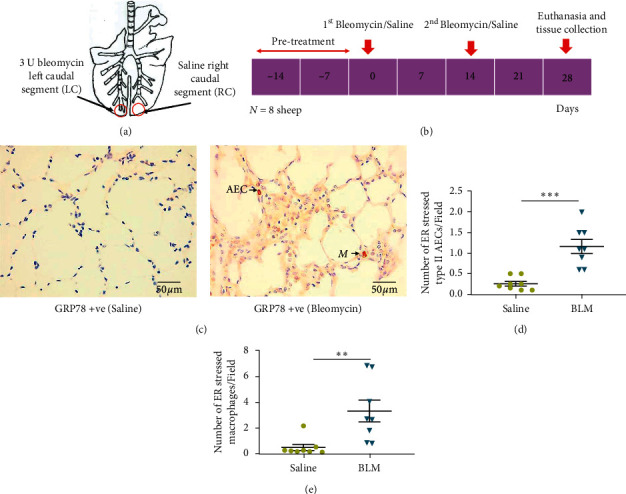
Schematic diagram of bleomycin infusion protocols in sheep (*n* = 8) and immunohistochemical staining against GRP78 to identify ER stressed type II AECs and macrophages in sheep parenchymal lung tissues. (a) In individual sheep (*n* = 8), the left caudal lung segment received infusions of bleomycin, while the right caudal lung segment received saline for internal control purposes. (b) Diagram shows the timeline for bleomycin/saline infusions and the tissue collections. These infusions were repeated at day 14 and tissue samples were harvested at day 28. All the animals were kept for 14 days acclimation period (pre-treatment) prior to bleomycin/saline administration. (c) Representative images of GRP78 immuno-stained sections taken from the differentially treated lung segments. Arrows indicate ER stressed type II alveolar epithelial cells (AECs) and macrophages (M). (d, e) Graphs represent the number of ER stressed type II AECs and macrophages in bleomycin- (BLM-) and saline-infused lung segments (*n* = 8 sheep). The data was taken from twenty representative, non-overlapping fields randomly captured under 40x magnification. Each bar represents the mean ± standard error of the mean. Significance was determined by *t*-test.  ^*∗∗*^*p* < 0.001 and  ^*∗∗∗*^*p* < 0.0001.

Pulmonary fibrosis was induced in eight female merino sheep (*n* = 8) aged within 9–12 months, using Bleomycin sulphate (Hospira, Melbourne, Victoria, Australia) prepared at a concentration of 0.6 U bleomycin/ml saline [25]. From the prepared bleomycin solution, 5 ml was administered as bolus into the desired lung segment via the biopsy port of the fiber-optic bronchoscope (total dose of 3 U bleomycin/lung segment). Similarly, 5 ml of 0.9% sterile saline was administered to the contralateral caudal lung segment as an internal control (Figure 1). The second dose of bleomycin/saline was administered to the same lung segments after 14 days (Figures 1(a) and 1(b)). Then, the sheep were kept another 14 days to develop lung fibrosis. All the sheep were sacrificed at day 28 by administering intravenous barbiturate (Lethabarb, Virbac Animal Health, Australia).

### 2.2. Necropsy and Tissue Sampling

Lungs were removed during the necropsy and the targeted lung segments were identified. Caudal segments of the targeted lung lobes were separated out and cannulated by exposing the bronchiole to inflate the segments with a 1 : 1 mixture of optimal cutting temperature (OCT) and sterile PBS solution under pressure of approximately 20 cm/H_2_O, in order to preserve the lung tissue architecture during processing. Then, 0.5 cm thickness lung tissue samples were fixed in 4% paraformaldehyde followed by 70% ethanol and processed in paraffin for histopathological analysis.

### 2.3. Evaluation of ER Stress in Type II AEC and Macrophages

Persistent injury to type II AECs and lung macrophages potentially induces ER stress, which in turn can progress to apoptosis. Glucose regulated protein (GRP) 78 is one of the best-characterized ER chaperone proteins and serves as a classic marker to evaluate ER stress. Immunohistochemistry was performed using a rabbit monoclonal anti-GRP78 primary antibody (Abcam (ab108615)) to evaluate ER stress in type II AECs and macrophages on paraffin lung sections. Each assay was performed with a negative control without adding the primary antibody.

### 2.4. Anti-GRP78 Immunohistochemical Staining

Paraffin sections were subjected to deparaffinization and rehydration by immersing them in xylene for three 5 min changes, followed by two 5 min changes of absolute ethanol, followed by 70% ethanol for 5 min. Antigen retrieval was performed using pre-heated citrate buffer (pH-6) heated for 15 min, and the slides were left to cool down for 10 min followed by PBS washing. Then, the slides were kept in 3% H_2_O_2_ for 10 min to block endogenous peroxides and rinsed thoroughly with PBS. Undiluted fetal calf serum (FCS) was added to each slide and incubated for 1 hour to block non-specific bindings with the antigen. Anti-GRP78 antibody (Abcam, ab108615) diluted 1 : 50 with FCS was added to each sample and incubated for 1 hour. Slides were rinsed gently with PBS and then EnVision^TM^ dual link system-HRP (Horseradish Peroxidase) (Dako, North America Inc., CA, USA) was applied and incubated for 30 min. To visualize the antigen-antibody reaction, Nova RED peroxidase substrate (Vector Laboratories Inc., CA, USA) was added to each sample and incubated for 3 min. Then, the samples were washed with distilled water to stop the reaction and counter-stained with Hematoxylin.

### 2.5. Evaluation of Apoptosis in Type II AEC and Macrophages

**Figure 2:**
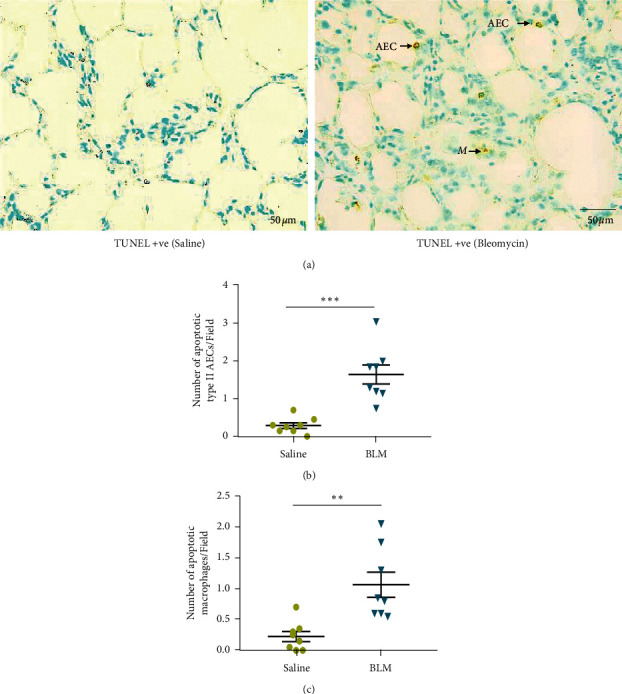
Click-iT™ TUNEL Colorimetric assay to identify apoptotic type II AECs and macrophages in sheep parenchymal lung tissues. (a) A representative photomicrograph, captured under 40x magnification for the evaluation. Arrows indicate apoptotic type II alveolar epithelial cells (AECs) and macrophages (M). Graphs represent the number of apoptotic type II AECs and macrophages between bleomycin and saline infusion at the fibrotic stage in sheep lung tissues (*n* = 8) (b, c). Each bar represents the mean ± standard error of the mean. The tissues were sampled from lung segments at post mortem as indicated in Figures 1(a) and 1(b). Significance was determined by *t*-test.  ^*∗∗*^*p* < 0.001 and  ^*∗∗∗*^*p* < 0.0001.

Click-iT^TM^ Colorimetric Terminal Deoxynucleotidyl Transferase-dUTP Nick End Labeling (TUNEL) assay (Click-iT^TM^ TUNEL Colorimetric IHC Detection Kit, Thermo Fisher Scientific, USA) was performed on paraffin lung tissue sections to evaluate type II AECs and macrophages turnover in bleomycin-induced lung fibrosis in sheep model (Figure 2). Each assay was performed with a negative control without adding TdT reaction mixture. Paraffin sections were deparaffinized and rehydrated by xylene and a graded series of ethanol according to the manufacturer's instructions (Click-iT^TM^ TUNEL Colorimetric IHC Detection Kit, Thermo Fisher Scientific, USA). The slides were dipped in 1% H_2_O_2_ for 10 min to block endogenous peroxidase. Diluted Proteinase K (dilution rate 1 : 50) was added to each sample and incubated for 5 min. Then, the samples were blocked with blocking buffer (1% Bovine Serum Albumin (BSA) + 5% Normal Sheep Serum (NSS) + PBS) for 30 min. Slides were washed thoroughly with PBS for 5 min 3 times prior to each step. For the Terminal Deoxynucleotidyl Transferase (TdT) reaction, slides were incubated with TdT reaction buffer for 10 min. TdT reaction mixture was prepared according to the manufacturer's protocol and added to each slide and incubated for 60 min. After rinsing with PBS to fully quench the TdT reaction, slides were then immersed in Saline Sodium Citrate (SSC) buffer for 15 min and then in PBS and click-iT TUNEL Colorimetric washing solution. Click-iT TUNEL Colorimetric reaction cocktail (Click-iT reaction buffer + CuSo4, biotin azide + reaction buffer additive) was prepared according to the manufacturer's recommendation, added to each sample, and incubated for 30 min. Samples were washed with PBS and Click Colorimetric washing solution. Then, the samples were incubated with Streptavidin-Peroxidase for 30 min followed by washing with PBS and deionized water. DAB was diluted 1 : 200 with DAB substrate buffer and added to each sample and kept for 30 seconds to facilitate the reaction. Then, the sections were washed with deionized water to stop the reaction and counter-stained with methyl green. 2 g of methyl green was dissolved in 100 ml of distilled water and extracted in chloroform to remove the crystal violet. Working solution was prepared by mixing 1 : 1 methyl green with 0.1 M acetate buffer. Slides were then counter-stained with methyl green for 10 min, followed by quick dehydration with 70% and 100% ethanol and fixed in xylene.

### 2.6. In Vivo Experimental Design to Evaluate the Therapeutic Effect of Senicapoc and Pirfenidone on the ER Stress and Apoptosis of Type II AEC and Macrophage in Bleomycin-Induced Lung Fibrosis

**Figure 3:**
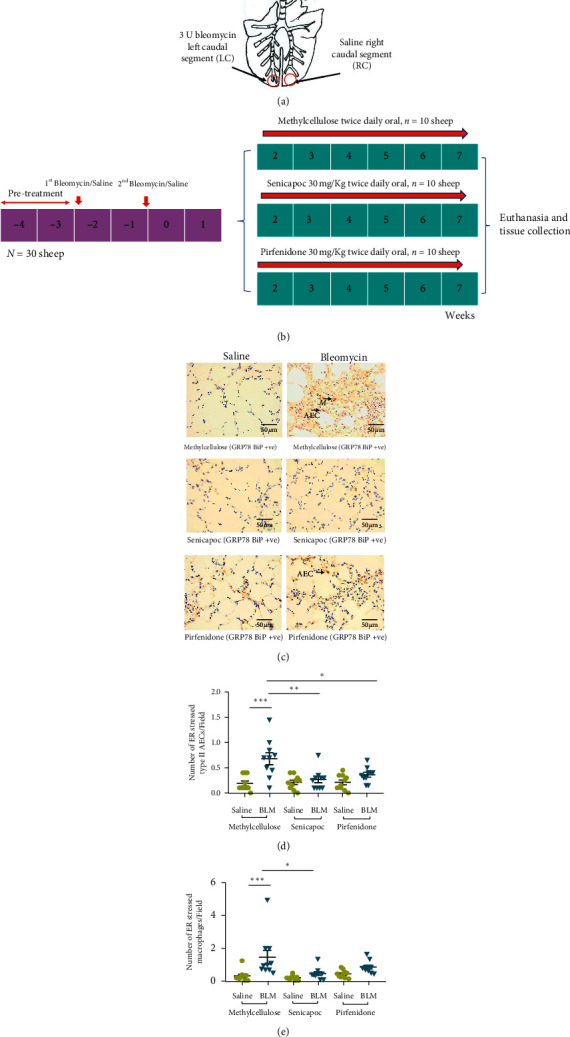
The effects of senicapoc and pirfenidone on ER stress induced by bleomycin in type II AECs and macrophages. (a) In individual sheep, the left caudal lung segment received infusions of bleomycin, while the right caudal lung segment received the saline. (b) Diagram shows the timeline of the bleomycin administration, treatment protocols (methylcellulose (control), senicapoc, pirfenidone) of the three groups of sheep, and the tissue collection. (c) Representative images from immunohistochemical staining against GRP78 to identify ER stress in type II AECs and macrophages in sheep lung segments treated with either methylcellulose (control), senicapoc, or pirfenidone. Arrows indicate ER stressed type II alveolar epithelial cells (AECs) and macrophages (M). (d, e) Graphs show the rate of ER stress in type II AECs and macrophages between the different treatment groups (*n* = 10 sheep/group). The data was collected from twenty representative, non-overlapping fields captured at 40x magnification. Each bar represents the mean ± standard error of the mean. Significance was determined by one-way ANOVA and a Tukey's post hoc test to make multiple comparisons test between groups.  ^*∗*^*p* < 0.05,  ^*∗∗*^*p* < 0.001, and  ^*∗∗∗*^*p* < 0.0001.

Sheep lung tissues were obtained from our previous studies [24, 26] and a brief description of the experimental design follows. Pulmonary fibrosis was induced in 30 merino sheep (*n* = 30) aged 9–12 months using bleomycin as described above (Figures 3(a) and 3(b)). The sheep were allowed to develop lung fibrosis for another 2 weeks. The sheep were then randomly grouped into three groups (*n* = 10). Each group was then treated with either 30 mg/kg senicapoc (Icagen Inc., Durham, NC)/pirfenidone (Selleckchem, USA) or methylcellulose (Control) 0.5% methyl-cell MC (Sigma-Aldrich, Castle Hill, NSW, Australia) orally twice daily. At the end of the treatment regime, sheep were sacrificed by administering intravenous barbiturate (Lethabarb, Virbac Animal Health, Australia) and lung tissues were collected and processed as mentioned above. ER stress and apoptosis of type II AECs and macrophages were evaluated by staining against anti-GRP78 and TUNEL assay, respectively, as mentioned above.

### 2.7. Quantitative Image Analysis

Images were captured using Leica DM500 microscope. Twenty representative non-overlapping fields were selected and captured under 40x magnification. Brown stained cells were counted in each field and expressed as means and standard errors of means (mean ± SEM). Type II AECs and macrophages were identified based on their morphology. Type II AECs are cuboidal cells with centrally located nuclei and the cell is covered by the basement membrane, with a small portion exposed to the alveolar surface [3]. Macrophages were identified by their large irregular shape with a prominent nucleus and granular cytoplasm.

### 2.8. Statistical Analysis

Statistical analysis was carried out using the software GraphPad Prism version 8.0.1 for Windows (GraphPad Software, La Jolla California USA). Normality testing was performed using D'Agostino-Pearson omnibus normality test. *t*-test was performed to evaluate ER stress and apoptosis in type II AECs and macrophages in sheep lung tissues. The effects of senicapoc and pirfenidone on ER stress and type II AECs and macrophage turnover were evaluated using one-way ANOVA with Tukey's post hoc test to make multiple comparison between groups. An observation with a *p* value less than 0.05 (*p* < 0.05) was considered statistically significant.

## 3. Results

### 3.1. ER Stress Precedes in Bleomycin-Induced Lung Fibrosis in Sheep Model

ER stress was assessed by determining the number of GRP78 immuno-positive cells in sections sampled from differentially treated lung segments collected at post mortem (Figures 1(a) and 1(b)). ER stress in type II AECs and lung macrophages was shown by red cytoplasmic staining, indicating elevated expression of GRP78 proteins in (Figure 1(c)). The rate of ER stress in type II AECs was significantly increased in sheep lung tissues two weeks after they were infused with bleomycin (bleomycin 1.16 ± 0.17 vs saline 0.27 ± 0.06 cells/field, *p* = 0.0002) (Figure 1(d)). Similarly, there was a significant increase in the rate of ER stress in macrophages with the bleomycin infusion when compared to control/saline lung segments (bleomycin 3.30 ± 0.84 vs saline 0.46 ± 0.24 cells/field, *p* = 0.006) (Figure 1(e)).

### 3.2. Evaluation of Apoptosis in Type II AEC and Macrophages

Extensive fragmentation of nuclear DNA due to activation of endonucleases is a hallmark of late-stage apoptosis. Fragmented DNA (TUNEL-positive) was indicated by brown nuclear staining in type II AECs and macrophages (Figure 2(a)). As shown in Figure 2(b), the rate of apoptosis in type II AECs increased significantly in bleomycin-infused lung segments when compared to saline-infused lung segments (bleomycin 1.64 ± 0.25 vs saline 0.28 ± 0.07 apoptotic cells/field, *p* = 0.0001). A significant increase was also observed in the number of apoptotic macrophages in the bleomycin-infused lung segments, when compared to the control lung segments (bleomycin 1.06 ± 0.20 vs saline 0.22 ± 0.08 apoptotic cells/field, *p* = 0.0001).

### 3.3. KCa3.1 Ion Channel Blockade Significantly Attenuates ER Stress and Apoptosis in Type II AEC and Lung Macrophages in Lung Fibrosis Induced by Bleomycin

The KCa3.1 ion channel has been shown to play a crucial role in the development of ER stress and apoptosis in type II AECs and lung macrophages [18]. Therefore, we investigated whether ER stress and apoptosis in type II AECs and lung macrophages can be reduced by blocking the Ca^2+^-activated K^+^ ion channel (KCa3.1) using the KCa3.1-specific ion channel-blocking drug senicapoc. In this experiment, we evaluated the ER stress and apoptosis in lung tissues from the respective groups 7 weeks after the final bleomycin infusion. The group structure and experimental timeline for procedures in this experiment are given in Figures 3(a) and 3(b).

Cytoplasmic expression of the ER stress marker GRP78 in macrophages and AECs is shown for the differentially treated lung segments in Figure 3(c). In control sheep treated with methylcellulose, there were increased numbers of GRP78-positive type II AECs (saline 0.19 ± 0.04 vs bleomycin 0.68 ± 0.12 cells/field, *n* = 10, *p* = 0.0003) (Figure 3(d)) and macrophages (saline 0.32 ± 0.10 vs bleomycin 1.46 ± 0.42 cells/field, *n* = 10, *p* = 0.005) (Figure 3(e)) in bleomycin-infused lung segments when compared to saline-infused segments. This indicated that the disease model was functioning correctly. When sheep groups treated with either senicapoc or pirfenidone were compared with the methylcellulose control group, the number of AECs expressing the GRP78 protein was significantly reduced in bleomycin-infused lung segments of sheep treated with either senicapoc or pirfenidone (senicapoc 0.26 ± 0.06 vs 0.68 ± 0.12 cells/field in vehicle, *n* = 10 *p* = 0.002; pirfenidone 0.36 ± 0.04 vs 0.68 ± 0.12 cells/field in vehicle, *p* = 0.01) (Figure 3(d)). Furthermore, in senicapoc and pirfenidone-treated sheep, the number of GRP78-positive AECs in bleomycin-infused lung segments was not significantly different to saline-infused lung segments (Figure 3(d)). These data show that the increase in ER stress in AECs due to bleomycin injury was attenuated with treatment with either pirfenidone or senicapoc.

For macrophages, only treatment with senicapoc significantly reduced the number of macrophages in ER stress in lung segments exposed to bleomycin, when compared to vehicle-treated control lung segments injured by bleomycin (senicapoc 0.47 ± 0.11 vs 1.46 ± 0.42 cells/field in vehicle, *p* = 0.017; pirfenidone 0.86 ± 0.11 vs 1.46 ± 0.42 cells/field in vehicle, *p* = 0.2) (Figure 3(e)). In senicapoc-treated sheep, the number of GRP78-positive macrophages in bleomycin-infused lung segments was not significantly different to saline-infused lung segments (Figure 3(e)). While the number of GRP78-positive macrophages in bleomycin-infused lung segments of pirfenidone-treated sheep was higher than saline-infused lung segments, the data was also not significantly different (Figure 3(e)).

**Figure 4:**
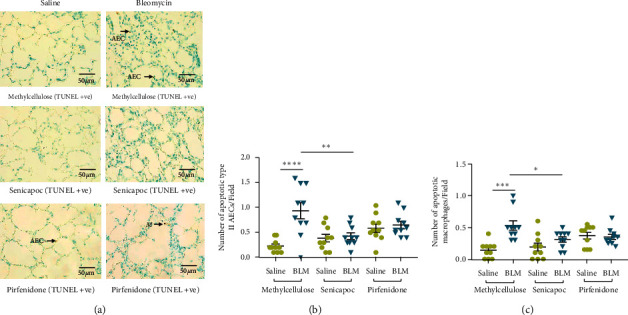
Effects of senicapoc and pirfenidone treatment on apoptosis induced by bleomycin in type II AECs and macrophages. (a) Representative images from colorimetric TUNEL assays to identify apoptotic type II AECs and macrophages in sheep lung segments treated with either methylcellulose (control), senicapoc, or pirfenidone. Arrows indicate apoptotic type II alveolar epithelial cells (AECs) and macrophages (M). (b, c) Graphs represent the rate of apoptosis in type II AECs and macrophages between the different treatment groups (*n* = 10 sheep/group). The data was collected from twenty representative, non-overlapping fields captured at 40x magnification. Each bar represents the mean ± standard error of the mean. The group structure and experimental timeline for procedures in this experiment are given in Figures 3(a) and 3(b). Significance was determined by one-way ANOVA and a Tukey's post hoc test to make multiple comparisons test between groups.  ^*∗*^*p* < 0.05,  ^*∗∗*^*p* < 0.001, and  ^*∗∗∗*^*p* < 0.0001.

TUNEL-positive fragmented DNA is presented as brown nuclear staining in type II AECs and macrophages for the differentially treated lung segments in Figure 4(a). In control sheep treated with methylcellulose, there were increased numbers of TUNEL-positive type II AECs (saline 0.23 ± 0.04 vs bleomycin 0.94 ± 0.16 cells/field, *n* = 10, *p* < 0.0001) (Figure 4(b)) and macrophages (saline 0.14 ± 0.04 vs bleomycin 0.53 ± 0.07 cells/field, *n* = 10, *p* = 0.0001) (Figure 4(c)) in bleomycin-infused lung segments when compared to saline-infused segments. This indicated that the disease model was functioning well and the apoptosis in type 2 AECs and macrophages remains high at 7 weeks after the final bleomycin infusion.

When sheep groups treated with either senicapoc or pirfenidone were compared with the methylcellulose control group, the number of TUNEL-positive AECs was significantly reduced in bleomycin-infused lung segments of sheep treated with senicapoc (senicapoc 0.43 ± 0.06 vs 0.94 ± 0.16 cells/field in vehicle, *n* = 10, *p* = 0.003) (Figure 4(b)). While the number of apoptotic AECs was lower with pirfenidone treatment, when compared with methylcellulose control treatment, the difference was not significant (pirfenidone 0.65 ± 0.07 vs 0.94 ± 0.16 cells/field in vehicle, *n* = 10, *p* = 0.2) (Figure 4(b)). In both senicapoc- and pirfenidone-treated sheep, the number of TUNEL-positive AECs in bleomycin-infused lung segments was not significantly different to saline-infused lung segments (Figure 4(b)).

For macrophages, treatment with senicapoc significantly reduced the number of apoptotic of macrophages in lung segments exposed to bleomycin, when compared to vehicle-treated control lung segments injured by bleomycin (senicapoc 0.31 ± 0.04 vs 0.53 ± 0.07 cells/field in vehicle, *n* = 10, *p* = 0.04; pirfenidone 0.35 ± 0.04 vs 0.53 ± 0.07 cells/field in vehicle, *n* = 10, *p* = 0.1) (Figure 4(c)).

## 4. Discussion

This study is the first to report that the bleomycin treatment increases ER stress and apoptosis in type II AECs and lung macrophages, and this effect is significantly ameliorated by *in vivo* blockade of the KCa3.1 ion channel with the drug senicapoc. Our previous studies using the sheep pulmonary fibrosis model reported that there were significant reductions in lung fibrosis and microvascular remodeling in sheep treated with senicapoc [23, 24]. The attenuated lung pathology in senicapoc-treated sheep coincided with improved lung function in these animals [24]. Findings of the current study show that blockade of the KCa3.1 ion channel reduces ER stress and apoptosis in type II AECs and macrophages, and this effect presumably contributes to the reduced pathology and improved lung function observed in this sheep model of pulmonary fibrosis.

Chronic ER stress-mediated apoptosis in type II AECs and macrophages has been identified as one of the principle pathogenic mechanisms in IPF [12, 27]. But the underlying mechanisms of ER stress-mediated apoptosis are complex and not yet fully understood [28]. Our results show that the prevalence of ER stress and apoptosis in type II AEC and macrophages was high 2 weeks after the final bleomycin infusion and remained at significantly high levels for at least 7 weeks after bleomycin injury.

Caution should be noted that these conclusions are based on relatively small numbers of apoptotic and ER stressed cells in the differentially treated lung samples. The range of ER stress-, or apoptotic-positive cells, observed in our study, was within 0–6 cells per field. Although statistically significant reductions in these indices were found for the drug treatments, the question that needs to be asked is whether these observed indices reductions have clinically relevant outcomes. Some of the low positive cell counts per field are due to the low overall density of cells in lung parenchyma, compared to other organs, resulting from the relatively high air-to-tissue-space ratio in the gas exchange region of the lung. In a previous study of lung idiopathic interstitial pneumonias, the frequency of TUNEL-positive cells in lung epithelium was a relatively low one to three percent of total lung epithelial cells counted compared to cells in culture [29]. The authors argue that apoptotic cells are rapidly phagocytosed and thus are only present for a relatively short time under light, or electron, microscopy. Thus, while the frequency of TUNEL-positive apoptotic cells visualized in tissue specimens is low, this small number may still reflect a considerable magnitude of cell loss [30]. Overall, the reductions in cellular apoptosis and ER stress observed with pirfenidone and senicapoc in the current sheep study should be seen in context with our previous clinical findings with these drugs. We have previously shown significant improvements in lung compliance, fibrotic scores, vascular remodeling, and collagen deposition in sheep treated with the KCa3.1 channel blocker senicapoc and the FDA-approved drug pirfenidone [23, 24, 26]. In the current study, the reduction of apoptosis and ER stress after treatment with these drugs is part of their overall clinical effectiveness.

The relationship between these intracellular events and the KCa3.1 ion channel needs to be further elucidated. We have reported that the KCa3.1 ion channel is widely expressed in alveolar epithelial cells [24]. Membrane potential and Ca^2+^ signaling of alveolar epithelial cells are mainly regulated by KCa3.1 channels in order to maintain critical cellular functions such as activation, migration, and proliferation [18, 31]. The KCa3.1 ion channel is a voltage-independent K^+^ channel that opens when intracellular Ca^2+^ levels increase, resulting in Ca^2+^-activated K^+^ efflux. This process plays an important role in regulation of cellular volume. When there is a continuous stimulus, the KCa3.1 ion channels become activated [18, 32]. This will result in substantial loss of intracellular K^+^ ions to the extracellular compartment, and more than 50% of the intracellular K^+^ can be lost via efflux [18]. Depletion of intracellular K^+^ ions induces apoptosis via caspase activation, cytochrome c release, and DNA degradation [33–35]. It is likely that the in vivo blockade of KCa 3.1 channels in the current study maintains ion balance between the intracellular and extracellular compartments, which in turn leads to a reduction of apoptosis [36].

The accumulation of unfolded proteins, imbalance of Ca^2+^ homeostasis, oxidative stress, and ischemia are some of the factors responsible for inducing ER stress in degenerative and fibrotic disorders in multiple organs [14, 37]. GRP78 is an ER stress chaperone protein that plays an important role in maintaining ER protein synthesis, folding, and intracellular calcium homeostasis [10, 38]. By evaluating the increased expression levels of GRP78 protein in type II AECs and macrophages, we demonstrated that the rate of ER stress in both type II AECs and macrophages was significantly retarded by blocking the KCa3.1 ion channel with senicapoc. Our findings are consistent with previous work evaluating the correlation between the calcium-dependent ER stress and KCa3.1 channels, where blockade or genetic deletion of the KCa3.1 ion channel was shown to reduce Ca^2+^ overload and attenuate ER stress in astrocytes of both humans and mice [39]. While these experiments were dealing with human and mouse brain cells, the findings are consistent with the reduction of bleomycin-induced ER stress in type II AECs and lung macrophages we observed in sheep treated with senicapoc.

We found that administration of the approved anti-fibrotic drug pirfenidone also reduced the rate of apoptosis in type II AECs and macrophages in bleomycin-infused segments. The underlying molecular mechanisms responsible for pirfenidone anti-fibrotic action are not fully understood. It is known that pirfenidone inhibits TGF-*β* stimulated collagen synthesis, myofibroblast differentiation, and fibrogenic activity of human lung fibroblasts [4–6]. Alveolar macrophages, type II AECs, myofibroblasts, and eosinophils are the main sources of TGF-*β* in lung fibrosis [40]. As TGF-*β* is a potent inducer of apoptosis of type II AECs, pirfenidone may exert its effect through suppression of the TGF-*β* pathway.

## 5. Conclusion

In vivo blockade with a specific inhibitor of the Kca3.1 ion channel alleviates the ER stress and apoptosis in type II AECs and macrophages. Our data provides an insight into how this drug interacts with these cell types in fibrotic lung tissues and thus contributes to the understanding of its anti-fibrotic effects in pulmonary fibrosis.

## Data Availability

The raw datasets in this study can be obtained from the corresponding author upon request.

## Abbreviations

IPF: Idiopathic pulmonary fibrosis
ER: Endoplasmic reticulum
AECs: Alveolar epithelial cells
KCa3.1: Calcium-activated potassium ion channel
GRP78: Glucose regulated protein 78
FDA: Food and drug administration
TGF-*β*: Transforming growth factor *β*
OCT: Optimal cutting temperature
PBS: Phosphate buffer saline
TdT: Terminal Deoxynucleotidyl Transferase
BSA: Bovine serum albumin
NSS: Normal sheep serum
TUNEL: Terminal Deoxynucleotidyl Transferase-dUTP nick end labeling
DAB: Diaminobenzidine
SEM: Standard error of means
LC: Left caudal
RC: Right caudal
BLM: Bleomycin.
